# Complications of Epicardial Pacing Wire Removal Following Adult Cardiac Surgery: A Systematic Review

**DOI:** 10.7759/cureus.49076

**Published:** 2023-11-19

**Authors:** Fathima S Mubarak, Yevinka Ellepola, Kadijah Nyosha Chamba, Sanjay Agrawal, Maged Makhoul

**Affiliations:** 1 Cardiothoracic Surgery, Harefield Hospital, Harefield, GBR; 2 Cardiac Surgery, West China School of Medicine, Chengdu, CHN; 3 General/Acute Medicine, Royal Devon and Exeter Hospital, Exeter, GBR

**Keywords:** retained hardware, delayed postoperative cardiac tamponade, surgery complication, pacing wires, adult cardiac surgery

## Abstract

Pacing wires are commonly used during cardiac surgery to monitor heart rhythm and, if necessary, provide temporary pacing. These wires are usually removed a few days after surgery, but the procedure has been associated with complications. The purpose of this study was to summarize the literature on complications related to pacing wire removal after cardiac surgery.

A systematic review was conducted using the PubMed, Embase, and Cochrane Library databases. Articles from January 1, 1998, to December 31, 2022, were considered. The literature was then registered with PROSPERO (registration number: CRD42023418165). PROSPERO is the first database to record systematic reviews in health, and it promotes best practices around the world through broad consultation to eliminate redundancy and waste of time and money. Following that, Preferred Reporting Items for Systematic Reviews and Meta-Analyses (PRISMA) was used to screen the data. PRISMA consists of a four-stage flow diagram and a "checklist" of 27 elements necessary for the rigorous and transparent dissemination of the systematic review's techniques and conclusions. These methods were used to ensure the integrity of the systematic review.

The systematic review included six studies with a total of 18,453 patients. The most common pacing wire removal complications were retention of the wire (0.56%), arrhythmia (0.67%), delayed discharge due to delayed wire removal (0.41%), and cardiac tamponade (0.1%). The overall complication rate was 1.74%. A subgroup analysis revealed that earlier removal (within 48-72 hours of surgery) was associated with a higher incidence of bleeding, whereas later removal (after 72 hours) was associated with a higher incidence of delayed discharge.

Pacing wire removal following cardiac surgery is associated with many complications, including retention of wire, arrhythmia, delayed discharge, tamponade, and death. These complications are more likely to occur with earlier or later removal of the pacing wires. Although the complication rate was lower, clinicians should be aware of these risks and take appropriate precautions when scheduling pacing wire removal. More research is needed to determine the necessity of pacing wires in cardiac surgery.

## Introduction and background

Epicardial pacing wires are routinely used during cardiac surgery in order to pace the patient momentarily. Pacing wires are mainly used to reduce atrioventricular (AV) blocks following adult cardiac surgery [1]. There could be perils in using epicardial pacing wires even though the wires are normally taken out a few days following surgery.

A review of complications related to epicardial pacing wire removal after adult cardiac surgery is necessary due to the potential hazards that can occur during and after the treatment. Complications can include hemorrhage, pericardial or mediastinal tamponade, cardiac dysrhythmias, wire fragment migration, and infection caused by residual wire fragments [2]. Due to these risks, patients are usually not discharged from the hospital until 24 hours after the procedure and may be hospitalized for up to five days [3]. Furthermore, knowledge of the complications can aid in the development of protocols and guidelines to improve patient outcomes and reduce the incidence of complications associated with the removal of epicardial pacing wires.

However, little in the medical literature is available to help standardize the use of epicardial pacing wires and to identify associated risks and benefits. While there is agreement among studies that complications may arise from epicardial pacing wire removal, certain questions remain open. For instance, there may be conflicting opinions on the appropriate timing for the removal of pacing wires in different hospital settings, which can lead to variations in decision-making practices among surgeons. Additionally, there may be disagreements on the best approach for managing specific complications that may arise during or after the removal of epicardial pacing wires [4].

The present systematic review aims to investigate the complications of temporary epicardial pacing wire (TEPW) removal and also to provide insight into the complications of wire removal in terms of timing.

## Review

Method

A predefined protocol was registered in PROSPERO (registration number: CRD42023418165). PROSPERO is a prospectively registered international database of systematic reviews in health and social care. The review protocol's key components are documented and kept as a permanent record. PROSPERO aids in preventing unintended duplication and enabling the comparison of reported review procedures with protocol expectations. As the first database to document systematic reviews in the health field, PROSPERO encourages best practices globally by facilitating widespread consultation to cut down on duplication and save costs and time.

This systematic review was written in accordance with the Preferred Reporting Items for Systematic Reviews and Meta-Analyses (PRISMA) statement [5]. PRISMA is a basic collection of items for publishing evidence-based systematic reviews and meta-analyses. PRISMA is primarily concerned with the reporting of reviews evaluating the effects of interventions, while it can serve as a framework for publishing systematic reviews with aims other than evaluating treatments. It is made up of a four-stage flow diagram and a "checklist" of 27 components that are required for the thorough and open communication of the methods and findings of the systematic review. This approach was employed to maintain the systematic review's integrity.

Potentially eligible studies were identified by searching the electronic MEDLINE and Embase databases through PubMed and Ovid, respectively. All studies that reported on epicardial pacing wires in adult patients were identified in the study selection. These databases were searched with keywords and Medical Subject Headings (MeSH) terms, "epicardial pacing wires," "complications of wire removal," and "adult cardiac surgery." Studies from January 1998 to December 2022 were reviewed in order to include more modern pacing wire technologies and changes in the recent cardiac surgical field.

All observational studies and case series with more than 10 patients were assessed for inclusion because there was a lack of randomized evidence. Pediatric cohorts, non-English research, and animal models were excluded. The removal of the epicardial wire was examined in specific case studies. Duplicate research, editorials, letters to the editor, comments, reviews, and meeting abstracts were excluded as well. The risk of mistake and publication bias was reduced through the use of pre-hoc sample size cutoffs. When securely removing epicardial wires, consideration was also given to the studies that did not reveal any complications to assess the apt timing of epicardial pacing wire removal without any complications.

The following key information was extracted from each publication: publication year, sample size, patient characteristics, surgery type, pacing wire removal timing, and complication reported. Discrepancies were resolved by discussion. The primary outcome is reported on complications of epicardial wire removal and subgroup analysis based on the timing of pacing wire removal.

Results

**Figure 1:**
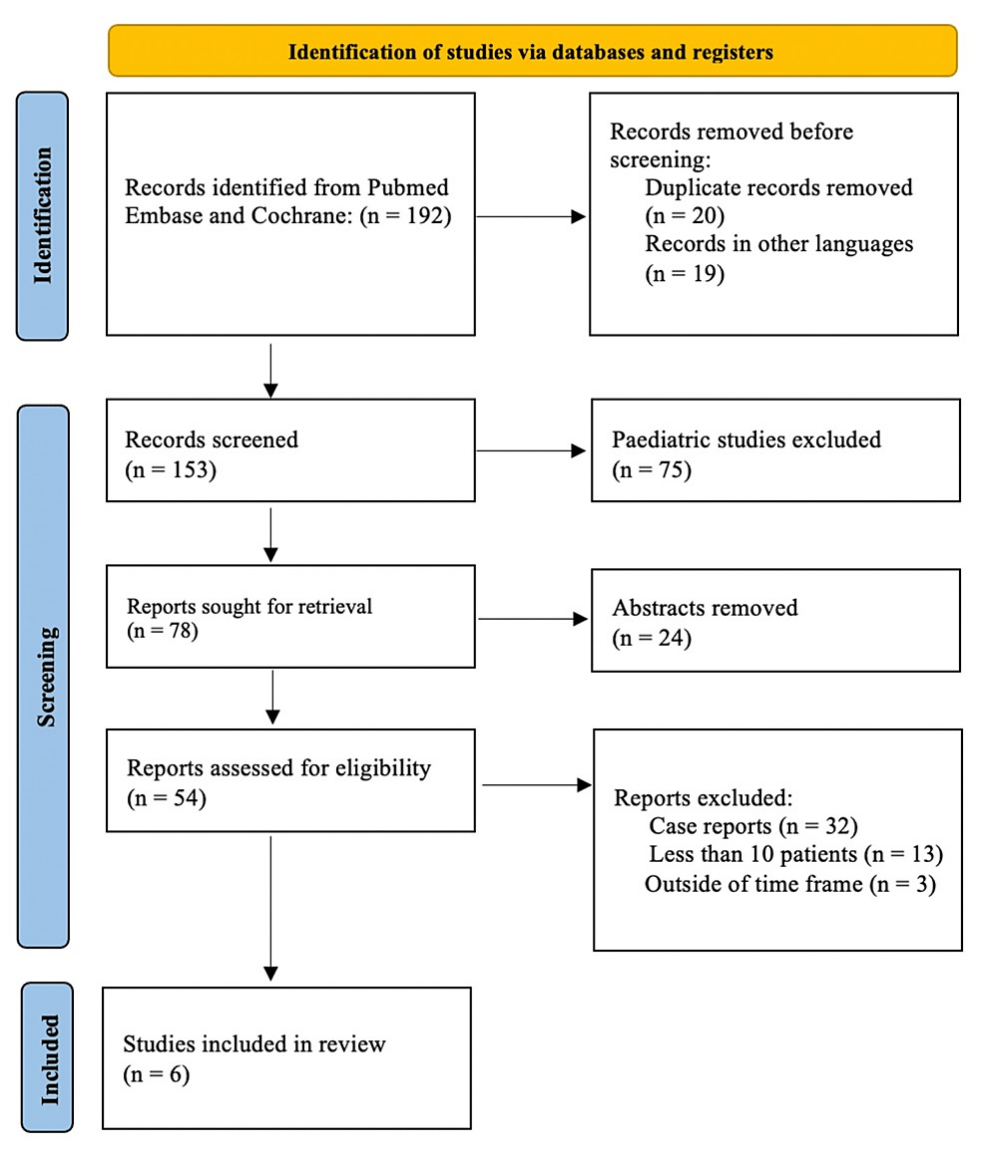
Study selection procedure shown in the PRISMA flow diagram PRISMA: Preferred Reporting Items for Systematic Reviews and Meta-Analyses

The predefined literature search yielded 192 results. Twenty studies were excluded due to being duplicates, and 19 studies were excluded based on their languages. Following a thorough review of the full texts, 147 studies were excluded for the reasons listed in Figure 1. Finally, six articles were selected for analysis (Figure 1).

The six articles were then reviewed for complications of epicardial pacing wire removal including 18,453 patients (Table 1).

**Table 1:** Study characteristics

Author	Study name	Sample size	Study design	Surgery type
Kiely et al. [3]	Epicardial pacing wires after cardiac surgery: an Irish cross-sectional study	164	Cross-sectional study	Coronary artery bypass graft/valve
Elmistekawy et al. [6]	Clinical and mechanical factors associated with the removal of temporary epicardial pacemaker wires after cardiac surgery	1,582	Prospective study	Coronary artery bypass graft/valve
Cote et al. [7]	Incidence of tamponade following temporary epicardial pacing wire removal	11,754	Retrospective study	Coronary artery bypass graft/valve
Carroll et al. [8]	Risks associated with removal of ventricular epicardial pacing wires after cardiac surgery	145	Prospective study	Coronary artery bypass graft/valve
Bougioukas et al. [9]	Is there a correlation between late re-exploration after cardiac surgery and removal of epicardial pacemaker wires?	4,244	Retrospective study	Coronary artery bypass graft/valve
Khorsandi et al. [10]	Is it worth placing ventricular pacing wires in all patients post-coronary artery bypass grafting?	564	Prospective study	Coronary artery bypass graft/valve

The most common pacing wire removal complications were retention of the wire (0.56%), arrhythmia (0.67%), delayed discharge due to delayed wire removal (0.41%), and cardiac tamponade (0.1%). The overall complication rate was 1.74% (Table 2).

**Table 2:** Complications of epicardial pacing wire removal

Complication	Incidence (number (%))
Retention of the wire	104 (0.56%)
Arrhythmia	124 (0.67%)
Delayed discharge	77 (0.41%)
Cardiac tamponade	19 (0.1%)
Overall complication rate	324 (1.57%)

Kiely et al. (2020) aimed to look into the use, duration, and consequences of temporary epicardial pacing wires implanted after heart surgery. According to the study, 74% of patients did not require pacing, and wires were routinely removed by day 4. Notably, after aortic valve replacement (AVR), 26% of patients were pacemaker-dependent, and 10% required lifelong pacemakers. Furthermore, the study found that while unneeded pacing wires are often removed on day 4, for a significant fraction of patients, they remained in place longer, and some faced delayed discharge (77) due to wire removal regulations [3].

Concerning clinical and mechanical factors associated with the removal of temporary epicardial pacemaker wires after cardiac surgery, one of the most serious consequences identified was bleeding, which required surgical intervention and could result in serious morbidity. The study emphasized the significance of meticulous TEPW removal to reduce the risk of problems, as well as the need for additional research in this area to improve patient safety and outcomes. Due to the resistance of wire removal, in the study by Elmistekawy et al., 104 patients had wire retention as a complication [6].

Cote et al. (2020) reported that among 11,754 patients who had cardiac surgery, 88 (0.75%) were readmitted to the operating room for bleeding and/or tamponade, and 11 (0.09%) of these cases were linked to TEPW removal. Notably, two of the tamponade patients sustained irreparable anoxic brain injury. All 11 patients were on antiplatelet therapy and/or anticoagulation, which is the institution's standard of care [7]. However, post-epicardial pacing wire removal arrhythmia was the only complication observed in the study. The risk associated with delayed removal of ventricular epicardial pacing wires after cardiac surgery was due to pacing dependency [8].

In the study "Is there a correlation between late re-exploration after cardiac surgery and removal of epicardial pacemaker wires?" by Bougioukas et al. (2017), in all instances, temporary epicardial pacemaker wires were inserted, and data on re-explorations for bleeding and pericardial tamponade were obtained, with an emphasis on late re-explorations, which occurred on the fourth postoperative day or later. Eight patients reported acute bleeding after the removal of temporary wires during late re-explorations for bleeding, accounting for 0.18% of cases [9]. Surprisingly, no complications were documented in the study "Is it worth placing ventricular pacing wires in all patients post-coronary artery bypass grafting?" by Khorsandi et al. (2012). Furthermore, this study suggests that, while ventricular pacing wires may play a role in the management of total AV block following coronary artery bypass grafting (CABG), routine implantation in all patients may not be necessary, and their usage should be tailored to individual patient risk factors and therapeutic justification [10].

**Figure 2:**
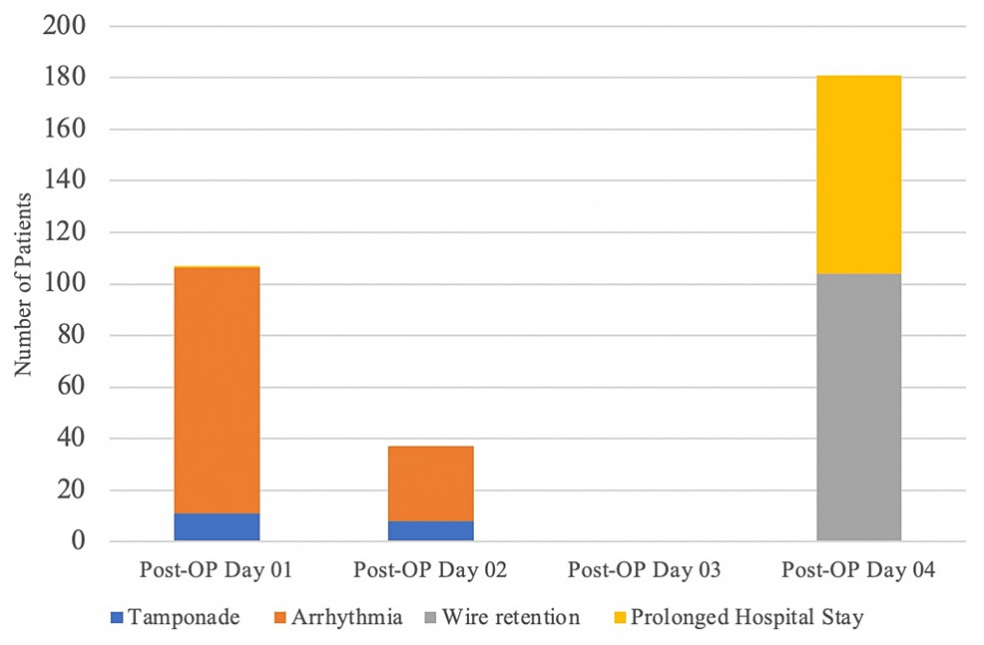
Complications according to the day of removal

A subgroup analysis revealed that earlier removal (within 24-48 hours of surgery) was associated with a higher incidence of bleeding, whereas later removal (after 72 hours) was associated with a higher incidence of delayed discharge (Figure 2).

Discussion

One of the major complications mentioned in the literature is cardiac tamponade, a medical emergency that develops when fluid collects in the pericardial sac, compressing the heart and decreasing cardiac output. In the examined studies, the prevalence of cardiac tamponade after TEPW removal was reported to be 0.1%. This highlights the importance of careful evaluation and management of individuals undergoing TEPW removal in order to avoid such significant consequences.

Bleeding and arrhythmias were also identified with TEPW removal. Bleeding can occur as a result of mechanical damage during the removal procedure and has been reported as a major side effect necessitating surgical intervention. Arrhythmias after TEPW removal might complicate the postoperative course, especially in patients who are pacing-dependent.

The timing of TEPW removal was found to be a significant factor influencing complications. Subgroup analysis indicated that earlier removal, within 24-48 hours of surgery, was associated with a higher incidence of bleeding, while later removal, after 72 hours, was associated with a higher incidence of delayed discharge.

Interestingly, the studies revealed variability in the utilization of TEPWs. While some patients benefited from temporary pacing wires, such as those who were pacer-dependent after aortic valve replacement (AVR); others did not require pacing and had the wires removed by day 4 post-surgery. This highlights the need for individualized patient care and tailored approaches to TEPW placement and removal.

The necessity of careful TEPW removal to lower the likelihood of problems was also underscored by the studies that were chosen. The interprofessional healthcare team's ability to coordinate effectively has been identified as a key factor in improving patient care and outcomes. The necessity of additional study in this field was also emphasized in order to improve procedures and patient safety.

A number of limitations should be recognized when considering this review, mainly pediatric cardiac surgery patients being excluded from this review. Many studies largely relevant to adult cardiac surgery were considered while creating this review. However, because the included studies were so diverse, not every outcome was documented in every publication. This makes evaluating the findings of a credible meta-analysis difficult; hence, only a systematic review was conducted. As a result, as illustrated by the degrees of variability, pooled rates should be evaluated with caution.

## Conclusions

The review and analysis of the chosen papers offer important new perspectives on the risks connected to temporary epicardial pacing wire removal following heart surgery. Potential side effects included cardiac tamponade, hemorrhage, arrhythmias, and delayed discharge. Additionally, recognized as significant influences on these issues were the timing of wire removal and the unique patient characteristics. To reduce the dangers connected with temporary epicardial pacing wire removal and improve patient outcomes, it is essential to do an accurate evaluation, coordinate with other healthcare practitioners, and conduct continuing research.
